# Abstract of the Proceedings of the Buffalo Medical Association

**Published:** 1857-11

**Authors:** Austin W. Nichols


ART. V.—*Abstract of the Proceedings of the Buffalo Medical Association.*

TUESDAY EVENING, Oct. 6, 1857.

The association met.

Present—The President, Dr. Flint, in the chair. Drs. Hamilton, Newman, Lemon, Hawley, Nichell, and Nichols.

The secretary being absent, the reading of the minutes of the last meeting was dispensed with.

Dr. A. W. Nichols was appointed secretary pro. tem.

There were no committees in readiness to report.

*Dr. Lemon* then read tne following report:

*Prostration and Coma, the effects of Fatigue, and Exposure to the Heat of the Sun.*

On Friday, the 17th July last, at noon, I saw Thomas Burns, aged 8 yrs. He was in a deep stupor; strong aqua ammonia being held to the nostrils made no impression, neither calling his name nor shaking would rouse him in the least.

The countenance was pale, the surface and extremities cold, the head about the natural temperature. Pulse 120, and quick respiration, labored but not stertorous.

His mother said he had been in the habit of playing about the docks and bathing frequently.

In the morning about 9 o'clock, he was seized with headache, vomiting and purging; these symptoms soon subsided and he gradually fell into the state of stupor described. Believing him to be laboring under the disease known as "sun-stroke," I treated him by means of the hot bath, ice to the scalp, sinapisms and brandy.

After having been in the bath about ten minutes he became conscious, moaned, opened his eyes, and struggled; brandy was then given, the patient removed to bed, wrapped in blankets, and mustard applied to the feet, insides of the thighs and to the epigastrium; ice pounded in a towel had been previously applied to his head, but soon removed.

In an hour from the time of his immersion, he could answer questions, protrude his tongue, and complained of the sinapisms. In two hours I left him comparatively comfortable.

The father told me next day, that the boy fell into a natural sleep soon after my departure, from which he awoke quite well. On that day, the thermometer, as noted at "Matthew's Dispensary," stood at 80° at noon.

There were several other cases in town the same week, one of which proved fatal. A St. Louis paper reports thirteen inquests held on the bodies of those having died from the effects of heat, on the 17th July.

The highest temperature this summer in Buffalo was on the 14th July, when the mercury rose to 93°.

In New York city, on the 14th of August, three men were prostrated in the afternoon, two died the same evening, the other lingered till next day. The thermometer ranged from 92 to 94°.

On that day at Augusta, Georgia, the mercury rose to 106° at noon.

Baltimore at noon, 94°.

Philadelphia, at 3, P. M., 94½°.

Albany in the afternoon, 97°.

Boston at mid-day, 92°.

The atmosphere has been moist, and the temperature lower on the average, than it has been for years before.—*Ext. from N. Y. Herald, August* 15, 1857.

The Daily Tribune for August 17th, reports nine deaths from "*coup de soleil*," August 14th and 15th.

Six were working on buildings, one in a sugar refinery, one a washerwoman, and one a collector of rents. All occurred in the afternoon, some died the same evening, others not until the same day. To the collector stimulants had been given, "Drs Mott and Moses" made a post-mortem examination, but the results are not given further than that in their opinion death was caused by exhaustion, the effects of heat and over exertion.

A business man on the docks has stated to me that he had often seen stevedores discharging cargoes, drinking freely of cold water, and perspiring copiously, suddenly become weak, sick, pale and cold, and lie down to sleep.

Great fatigue seems generally to precede an attack of "*solar exhaustion*," as a body of soldiers on a long march, laborers discharging cargo, sailors, &c., &c.

"A body of soldiers, mostly recruits, during a long march, were exposed to the rays of an East Indian sun; towards evening men were seen to drop down and immediately expire; others less severely attacked, were saved by timely and copious bleeding; but notwithstanding every exertion, at the close of the day, eighteen had died and sixty-three were sick.

"The loss, with one exception, was confined to the recruits, as was also all the sickness that followed.

"No examinations were made after death.

"In the beginning patients complained of dyspnœa, tightness and oppression about the chest, followed by giddiness; burning heat of the eyes; sense of fullness about the head, in many amounting to excruciating pain, succeeded by loss of sense and motion; faltering of the tongue in attempting to speak; dilated and fixed pupils; violent twitching of the muscles of the face; subsultus tendinum; involuntary stools; strong, full, and frequent pulse; tremendous throbbing of the carotid and temporal arteries; flushed, swollen, and sometimes livid countenance; and throughout a parched and burning skin.

"The treatment consisted of profuse depletion, blood being abstracted from the arm jugular vein and temporal artery; cold and blisters to the head; hot water foot-baths; calomel and antimony.

"Venesection was carried 'ad deliquium,' or to the relieving of the patient.

"It was found sometimes from fifty to one hundred ounces were required to produce this effect, and that sometimes the remedy was worse than the disease, for though the first attack might be relieved, yet subsequent symptoms required the same profuse evacuations, and from previous loss of blood the patient was not in a condition to bear such depletion. In fact, two individuals became convulsed, and died shortly after they were bled.

"After death it was found that although the heart was empty, the vessels of the head were loaded with blood. After this, the head was shaved, the body being wrapped in blankets, and no blood being abstracted until the pulse rose, and general heat and reäction had ensued."—*Extract from a Madras Medical Journal.*

We may, perhaps, be warranted in assuming the existence of two forms of the disease, the conditions being opposite, but produced by the same influences, governed by the physical state of the subject attacked.

In one case we may have congestion of the brain, and coma from pressure, fullness of the vessels, and general heat of the surface.

In another, all the conditions are opposite, the patient being in a state of collapse, hence the necessity of discrimination in respect to treatment in this as in any disease which may assume the sthenic or asthenic form.

*Prof. Hamilton* presented a specimen of *chronic rheumatic arthritis* occurring in the thigh-bone of a cow, which had been sent to him by Dr. Crosby, of Dartmouth, N. H. It has all of the characteristic appearances of this disease as we see it in the human subject. The head of the bone is flattened and expanded, and at certain points highly polished. The neck is also shortened and expanded; and around the neck, close to the trocanters, there is a large exostotic deposit. He was not aware that this disease had ever been observed before in other animals than man. In man it is not exceedingly rare. It occurs in persons who are advanced in life, either as an idiopathic affection, or as a consequence occasionally of a fall upon one of the trocanters, and for this reason it is occasionally mistaken for a fracture of the neck of the femur; and especially since it is followed by a gradual shortening of the limb, as in certain examples of intra-capsular fractures, we shall recognize the person who is suffering with this malady by his peculiar walk. It is a waddle. He moves, or rotates his body from side to side, in progression, because there is very little motion in the hip-joint.

Prof. H. thinks the disease is a chronic inflammation of the bone, and that the head and neck becoming softened, assume this abnormal shape in consequence of the pressure of the body upon the head. There may be some amount of interstitial absorption to explain the shortening of the neck, but it is quite as probable that the shortening is in a great measure a result of mere mechanical pressure, acting upon the bone when it is softened.

Prof. H. related some experiments he had made on chickens, in reference to the bending of bone. There had been, and was still, a difference of opinion in regard to the bending of bone; some surgeons, as Dupuytren, Barton, and Norris, regarded the bending of bone as a not uncommon occurrence. Dr. H. thinks that in all cases of alleged bending of bones, there is to a greater or less extent, interstitial fracture; or a fracture of some of the fibres within the bone.

The tibiæ of fourteen chickens, five or six weeks old, were bent to an angle of at least twenty-five degrees. In nine, there occurred no crack on bending, and in each case the bone resumed its place immediately. The inference from this experiment, making due allowance for the differing circumstances of the human bone and the bone of the bird, was that *bent bone* would resume its place immediately. It is to be observed that in these nine instances, no mark remained, nor any node or swelling existed. Dissection was made in from three to ten days. In no instance could Dr. H. detect any injury, except a small blood clot in the centre of the bone in a few cases.

The remaining five bones cracked when bent, and only one resumed its place. In two or three days thereafter, a distinct node-like swelling was felt. In dissecting the bones, from three to ten days after the partial fracture, the point of fracture was palpable; there was ensheathing callus at the seat of fracture. Dr. H. infers that if a bone, being partially broken, straightened spontaneously, it would leave indications of fracture. In those bones which were bent only, no sign of fracture was felt. Malgaigne admits that bones will bend and *remain* bent; but there must be a parallel bone broken completely to hold them. If no such broken bone held them or there was no partial fracture, the bent bones would resume their places. Dr. H. agrees with Malgaigne in this opinion. There are specimens of *bent bone*, but they occurred under the above circumstances.

The specimen which resumed its place after cracking, illustrates a class of cases which are found in the human subject. Although the axis of the bone is straight, there is a node-like swelling found. It is a partial fracture of the bone. Malgaigne and Blandin are the only authorities who have referred to this fact. In Dr. H.'s report to American Medical Association, were related cases of partial fracture of clavicle, in which the line of axis was immediately restored. The bone was partially broken, as the node-like swelling proved. This happened in the case of the clavicle in one out of every four cases. In these experiments, in one out of every five cases. The harmony between his observations in practice and in these experiments, is striking and should go far to establish the correctness of the opinions advocated above. A more more detailed account of these experiments will be found in the New York Jour. of Med, for Nov., 1857.

Dr. H. stated that in the above cases there was not much displacement; nor is there at any time much displacement of bone when broken by muscular action. In all the instances of bending merely, the chickens did not walk lame.

*Dr. Nichols* remarked that these experinents tended to confirm the opinion that bending of bone without fracture, was almost impossible, at least very rare, in the human body. The bone of birds differed essentially from that of the infant even, and in all cases of bending in which the bone did not resume its place immediately, there would be in almost every point of view, a greater likelihood to find fracture in the human subject, than in the bones of the chicken.

*Prof. Hamilton* farther stated, that no person has shown a specimen of *bent bone*, except under the circumstances above related; they are *always* cracked on one side.

*The President* suggested the propriety of admitting medical students to the meetings of the association.

*Prof. Hamilton* introduced the following resolution, which was carried without opposition:

"Resolved, That members be permitted to invite medical students to be present at the meetings of the association, but not to take part in the transactions."

And the association then adjourned.

AUSTIN W. NICHOLS,
Secretary *pro tem.*

